# Computational solvation analysis of biomolecules in aqueous ionic liquid mixtures: From large flexible proteins to small rigid drugs

**DOI:** 10.1007/s12551-018-0416-5

**Published:** 2018-04-23

**Authors:** Veronika Zeindlhofer, Christian Schröder

**Affiliations:** Faculty of Chemistry, Department of Computational Biological Chemistry, University of Vienna, Währingerstr. 17, Vienna, Austria

**Keywords:** Ionic liquid, Biomolecule, MD simulation

## Abstract

Based on their tunable properties, ionic liquids attracted significant interest to replace conventional, organic solvents in biomolecular applications. Following a Gartner cycle, the expectations on this new class of solvents dropped after the initial hype due to the high viscosity, hydrolysis, and toxicity problems as well as their high cost. Since not all possible combinations of cations and anions can be tested experimentally, fundamental knowledge on the interaction of the ionic liquid ions with water and with biomolecules is mandatory to optimize the solvation behavior, the biodegradability, and the costs of the ionic liquid. Here, we report on current computational approaches to characterize the impact of the ionic liquid ions on the structure and dynamics of the biomolecule and its solvation layer to explore the full potential of ionic liquids.

## Introduction

Ionic liquids (IL) are a unique class of high-performance chemical compounds with many applications in electrochemistry (Armand et al. 2009), synthesis (Itoh 2017), catalysis (Pârvulescu and Hardacre 2007; van Rantwijk and Sheldon 2007) as well as solvation (Hallett and Welton 2011) and extraction processes (Ventura et al. 2017). Their tunable properties via variation or modification of either the cation or the anion as well as their shared properties such as low vapor pressure, low flammability, and high thermal and electrochemical stability make them interesting solvents for various applications and started a hype in the beginning of the twenty-first century.

**Fig. 1:**
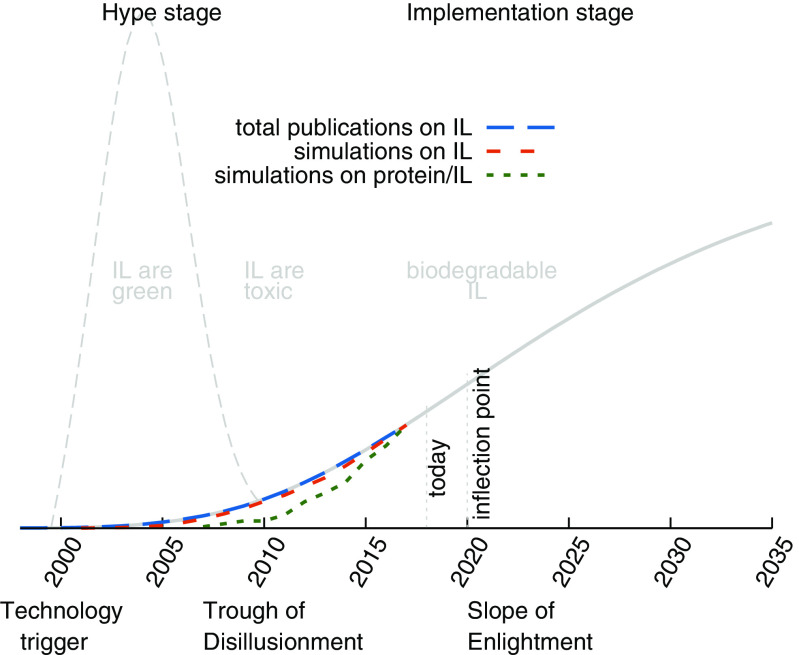
Gartner hype cycle on ionic liquids adapted from Kunz and Häckl (2016), Wasserscheid (2017)

Kunz and Häckl (2016) as well as Wasserscheid pointed out that the expectations followed a Gartner cycle as visible in Fig. 1. The initial success of ionic liquids in (bio-) catalysis (van Rantwijk and Sheldon 2007) and solvation (Welton 1999; Gutowski et al. 2003), e.g., dissolution of cellulose (Swatloski et al. 2002), served as a technology trigger. The vast number of $10^{18}$ possible combinations of cations and anions (Holbrey and Seddon 1999) seemed to promise the design of an optimal solvent for a particular application. As a result, the term “designer solvent” was coined (Freemantle 1998) and soon joined by the rash promise “ILs are green solvents” (Earle and Seddon 2009) leading to an exponential number of paper submissions to the journal *Green Chemistry*. Also, the availability of ILs increased due to beginning industrial production (Plechkova and Seddon 2008).

However, soon, first doubts were cast on the environmental benefit of ILs (Thuy Pham et al. 2010) and problems on the hydrolysis of some ILs were reported (Steudte et al. 2012). Additionally, many ionic liquids did not have a unique selling point or their improved performance did not justify their increased cost (Plechkova and Seddon 2008; Kunz and Häckl 2016). As a result, the hype cooled down in the past decade reaching the so-called Trough of Disillusionment of a Gartner cycle.

Nevertheless, scientific research did not abate, visible by the roughly exponential increase in publications indicated by the blue dashed line in Fig. 1. Here, we normalized the publication growth obtained from a search in the “Web of knowledge” to the current number of publications up to 2017. This way, we can compare the development with publication efforts concerning simulations of ionic liquids shown as an orange dashed line in Fig. 1 which agrees almost quantitatively with the overall publication output on ILs.

Quite generally, hype cycles can be decomposed into two processes (Sasaki 2015): a hype stage and an implementation stage. The latter can be fitted by an S-shaped Gompertz function

1 $$ f(t) = a \cdot e^{-e^{-k (t-t_{0})}}. $$

Taking the publication efforts depicted in Fig. 1 as a measure of implementation, the “Slope of Enlightenment” phase can be extrapolated. In addition to the amplitude *a* and the stretching factor *k*, the inflection point $t_{0}$ marks the year in which the implementations start to level off. In the case of the ILs, the year 2020 is obtained for both the total publications and the publications concerning the simulation of ionic liquids. The “Plateau of Productivity” will be reached in twenty to thirty years based on the current data.

In order to get there, many scientific questions have yet to be answered. In particular, the knowledge gained by simulations on biomolecules in ionic liquids and their mixtures is lagging behind the general ionic liquid trend as visible by the green dotted line in Fig. 1. The current review tries to summarize the current efforts of biomolecular solvation in ionic liquids and their mixtures from a molecular dynamics (MD) perspective.

**Fig. 2:**
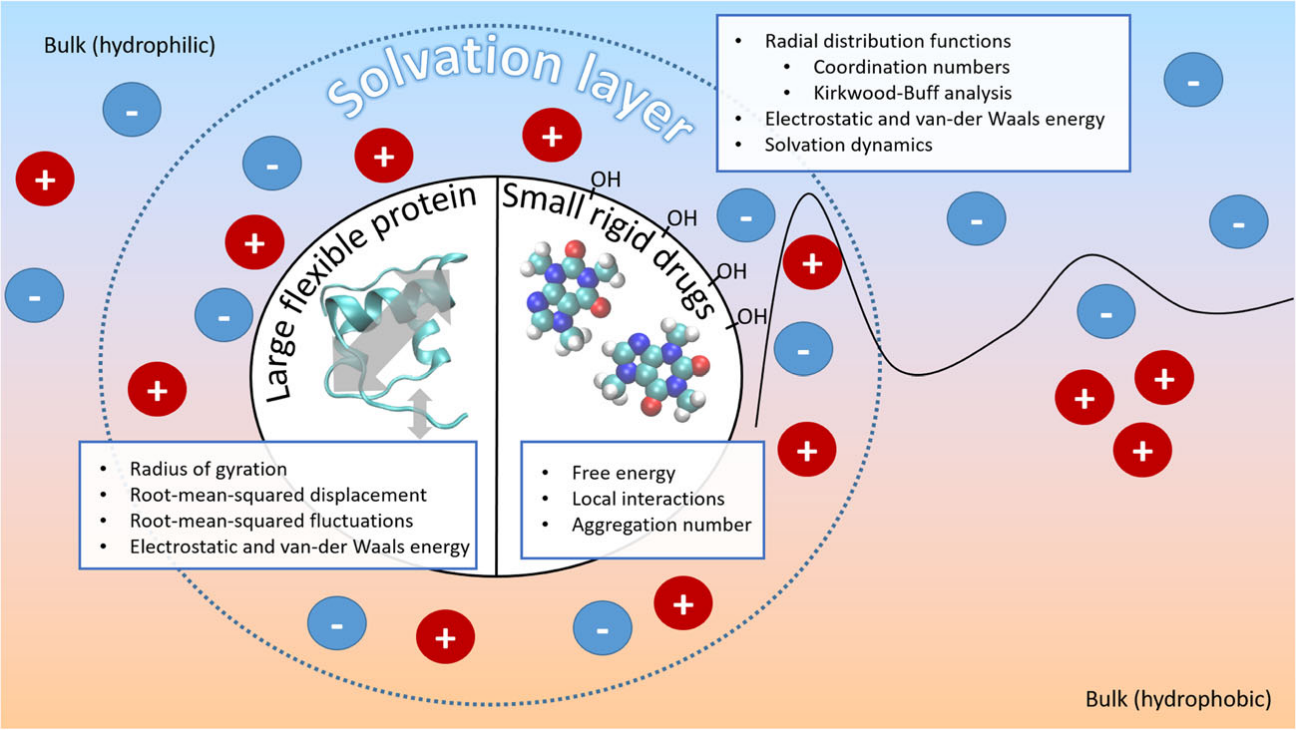
Computational analysis of the solvation interaction between biomolecules, their solvation layers, and the bulk solvent

Solvation starts at the first solvation layer which is the transition region between the bulk solvent and the solute as depicted in Fig. 2. Here, coordination numbers of particular solvent molecules and hydrogen bonding are interesting features to be computed from MD trajectories. Moreover, radial distribution functions (solid black line in Fig. 2) detecting the accumulation or depletion of solvent species at the biomolecular surface and their interpretation in terms of Kirkwood-Buff theory (Lesch et al. 2015; Diddens et al. 2017; Smiatek 2017) are reported in “The solvation layer”.

Due to the surrounding hydrophilic or hydrophobic solvent, the structure and stability of the biomolecular solute may adapt to the environment. Depending on the size and shape of a solute, different mechanisms contribute to solvent-induced changes. Hence, we distinguish between large flexible biomolecules and small rigid drugs, employing different analysis routines as sketched in Fig. 2:

1. Large proteins, polypeptides, or polymeric biomolecules possess a secondary structure which can deform during the solvation process (indicated by the gray arrows in Fig. 2). In a hydrophilic environment, for example, hydrophilic subunits are supposed to be at the surface of the biomolecule and in contact with the solvent. Hydrophobic subunits are covered in the core of the biomolecule. In addition to refolding, the size of a biomolecule may change when moving from a hydrophilic to a hydrophobic environment or vice versa. Computational size and shape observables of this biomolecular class are discussed in “Large flexible proteins”.
2. In contrast, small rigid drugs cannot increase their size or change their shape significantly. Here, other mechanisms like solute aggregation can be monitored by molecular dynamics. The inclusion of the solute in the solvent network can also be computed by free energy calculations. Furthermore, functional groups and their respective influence on the solvation are of significant importance. This will be discussed in “Small rigid drugs.”

## The solvation layer

The tunable solvation properties (Anderson et al. 2002; Gutowski et al. 2003) of ILs gave reasons for the hype on ILs at the start of this century. They were considered as green replacements for organic solvents in general (Cull et al. 2000) and for biological processes in particular (Yang and Pan 2005; Park and Kazlauskas 2003). For example, Rogers and co-workers reported on the dissolution of cellulose in 2002 (Swatloski et al. 2002). In 2005, BASF licensed the exclusive use of various intellectual property rights from this group. In 2007, the Rogers group continued their research on dissolution of lignocellulosic materials (Fort et al. 2007). Mikkola et al. (2007), Anugwom et al. (2012), Raut et al. (2015), Kilpeläinen et al. (2007), King et al. (2011), and Parviainen et al. (2013) worked on cellulose processing. Excellent reviews summarize the current development (Pinkert et al. 2009; Brandt et al. 2013; Mäki-Arvela et al. 2010; Zhang et al. 2017) and show that we are still in the “Slope of Enlightenment” phase during the implementation stage.

Due the high cost of ILs compared to the conventional alternatives, not many large-scale applications were introduced. In order to reduce the costs, ILs were mixed with other cheap solvents, in particular water. Since only few proteins are soluble in pure ionic liquids (Shao 2013; Kragl et al. 2002), homogeneous and heterogeneous aqueous ionic liquid mixtures were used (Kragl et al. 2002). The first ionic liquid-based aqueous biphasic system consisted of 1-butyl-3-imidazolium chloride mixture in aqueous K_3_*PO*_4_ and was reported by Rogers and co-workers in 2003 (Gutowski et al. 2003) and may be considered as a “Technology Trigger” (see Fig. 1). Recently, biphasic systems regained interest, in particular by the group of Coutinho for extraction (Ventura *et al*. 2009, 2017; Pei *et al*. 2009; Dreyer and Kragl^2008^; Pereira *et al*. 2010; Lee *et al*. 2017; Cláudio et al. 2010) and for purification of biomolecules (Pereira et al. 2013). An excellent review was given by Freire et al. (2012).

Although of great importance for the rational choice of an ionic liquid for any given application (Weingärtner et al. 2012), predicting the effect of an ionic liquid on the stability of a specific protein still remains difficult (Tung and Pfaendtner 2016) due to the complex and little understood molecular interactions between ionic liquid and protein (Burney et al. 2015) demonstrating that we are still in the “Slope of Enlightenment” phase and have not reached the “Plateau of Productivity” in Fig. 1. A popular classification of the effect of ionic liquids on proteins already mentioned is the extension of the well-known Hofmeister series (Zhang and Cremer 2006) to aqueous ionic liquid solutions (Constantinescu et al. 2007). The Hofmeister series ranks ions by their specific ion effect on protein properties such as stability or solubility (Zhang and Cremer 2006; Schröder 2017). Ions are classified either as kosmotropes (structure-makers) or chaotropes (structure-breakers) by their ability to influence water structure (Tung and Pfaendtner 2016; Constantinescu et al. 2007; Zhang and Cremer 2006). Generally, kosmotropic anions stabilize proteins, while chaotropic anions lead to destabilization (Tung and Pfaendtner 2016). Still, exceptions to the Hofmeister series have been observed (Zhang and Cremer 2009), not only due to the fact that specific protein properties such as charge and surface characteristics (Schröder 2017; Constantinescu et al. 2007; Tung and Pfaendtner 2016) are not considered. Moreover, the ability of ions to influence bulk water structure in a kosmo- or chaotropic manner has been questioned (Omta et al. 2003).

Less publications exist on the computational research of aqueous ionic liquid mixtures (Bhargava and Klein 2009; Varela et al. 2015; Chang et al. 2010) without additional solutes as these studies focus on more fundamental properties of IL mixtures rather than a particular application. The effect of water on the viscosity of an IL/water mixture was studied by Kelkar and Maginn (2007). Furthermore, the alkyl chain length of the IL cations changed the collective structure of the aqueous IL mixture (Bhargava and Klein 2009). We started our computational analysis on the collective network between water and various ionic liquids in 2007 (Schröder et al. 2007, 2008, 2009, 2014) with a focus on dielectric properties. Smiatek and co-workers published interesting papers on Kirkwood-Buff analysis of protein solvation in IL/water mixtures (Diddens et al. 2017; Smiatek 2017). Gupta and co-workers reported MD simulations on the interaction of cellulose with ionic liquids (Gupta et al. 2013). As a complete review of computational analysis of interactions of biomolecules with IL mixtures is out of the scope of this review, we will focus on the basic techniques to encourage other authors to contribute to the understanding of biomolecular solvation in ionic liquid mixtures by MD simulations . Hereafter, we will give a compact overview of solvation layer properties obtainable by MD simulations as well as their interpretation.

### Spatial distribution of the solvent

The spatial distribution of molecular species *j* around a reference site *i* is often characterized by the radial distribution function $g_{ij}(r)$,

2 $$ g_{ij}(r) = \frac{ \sum\limits_{j} \delta(r-r_{ij})}{4 \pi r^{2} dr \cdot} \cdot \frac{1}{\rho_{j}} $$

which is the ratio between the local density of *j* in a spherical shell $4 \pi r^{2} dr$ and the global density $\rho _{j}$ of species *j*. The distance $r_{ij}$ is usually defined between the center of masses of the molecules *i* and *j* or between the coordinates of particular atoms belonging to these molecules.

#### Coordination numbers

To calculate the coordination number $n_{ij}$ of species around the biomolecule *i*, spherical integration of the radial distribution function is commonly used:

3 $$ n_{ij} = \int\limits_{0}^{R_{1}} 4 \pi r^{2} g_{ij}(r) dr  $$

Micaêlo and Soares found that the coordination number of water around cutinase in aqueous 1-butyl-3-methylimidazolium nitrate [C_4_mim][NO_3_] and 1-butyl-3-methylimidazolium hexafluorophosphate [C_4_mim][PF_6_] mixtures is reduced at the protein surface (Micaêlo and Soares 2008). Similar removal of water was observed for hydrated *Candida rugosa* lipase 1 in [C_4_mim][PF_6_] and [C_4_mim][NO_3_] (Burney and Pfaendtner 2013).

A problem that may arise when comparing coordination numbers of different IL ions is the fact that the coordination number depends on ion density. For the analysis of cation and anion coordination numbers around CAL-B, Klähn et al. (2011a) normalized the coordination number by the average number of the respective species within 10 Å of the protein to facilitate comparison. They observed the highest ion densities around charged residues. Interestingly, although density of cations and anions was highest around oppositely charged residues, densities around residues with the same charge was also higher than for polar and non-charged residues (Schröder 2017; Klähn et al. 2011a), which is an effect of the strong interionic interactions in ionic liquids (Klähn et al. 2011b). Furthermore, a diffusion of cations into the active site of the protein occurred, possibly hindering substrate access. A similar occupation of the active site by cations was observed in simulations of xylanase II from *trichoderma longibrachiatum* in aqueous [C_2_mim][EtSO_4_] and [C_2_mim][OAc] (Jaeger and Pfaendtner 2013).

Steinhauser and co-workers noted that Eq. 3 not only requires a meaningful definition of a solvation shell radius $R_{1}$ but also assumes sphericity of the solutes (Haberler and Steinhauser 2011; Haberler et al. 2011; Zeindlhofer et al. 2018). Although this approach is feasible for many small solutes, it may give highly inaccurate results in case of anisotropic solutes (Zeindlhofer *et al*. 2017, 2018) like large proteins (Neumayr et al. 2010) due to non-symmetric excluded volume effects. Furthermore, for heterogeneous solvents with different molecular sizes, more than one shell radius would be necessary to describe a solvation shell appropriately (Haberler et al. 2011).

#### Voronoi-based coordination numbers

**Fig. 3:**
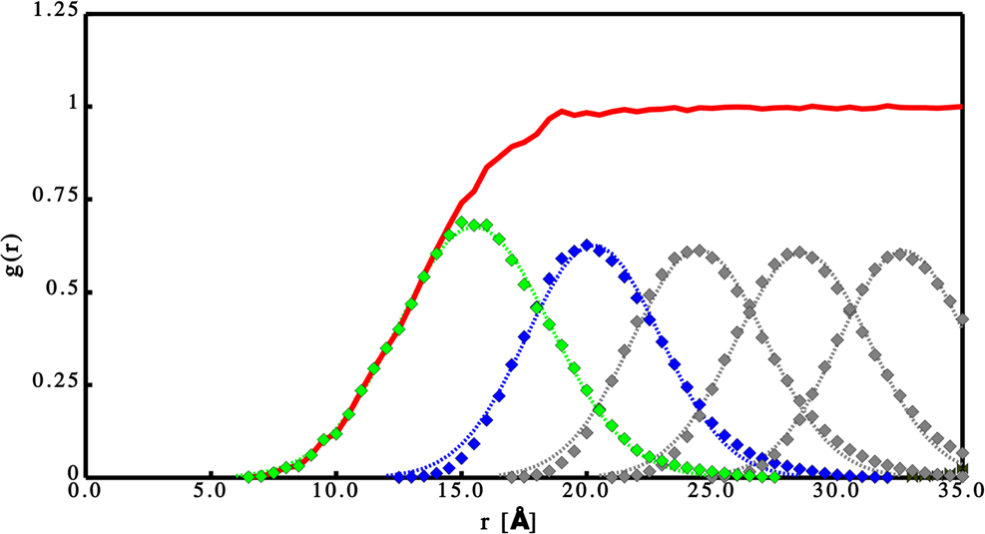
Radial distribution function *g*_*ij*_(*r*) of water oxygens (*j*) around the center of mass of calbindin *i* and the corresponding decomposition into Voronoi shells. Reproduced from Neumayr et al. (2010) with permission of AIP Publishing

An alternative method of spatial decomposition is Voronoi decomposition (Schröder et al. 2009). It is parameter-free and also allows for straightforward decomposition of multiple solvation shells. In Voronoi tessellation, each point in space representing an atomic coordinate is assigned an irregular polyhedron that contains all space closer to its associated reference point than to any other point in the system. If two points share a polyhedron face, they are defined as neighbors. Solvent molecules that share a face with the biomolecule are considered first-shell members, molecules that share a face with first-shell members are considered second shell, and so on. This analysis can be performed for each time step during a trajectory to yield mean residence times of particular species at the surface of the biomolecule (Haberler and Steinhauser 2011; Haberler et al. 2011). This shell assignment can also be applied to the radial distribution function (Neumayr et al. 2010; Zeindlhofer et al. 2018) as depicted in Fig. 3. The decomposition of the displayed $g_{ij}(r)$ into spherical solvation shells discussed in the last section is not possible as no clear first minimum of the radial distribution can be detected. Furthermore, the different shells overlap to a large extent. Consequently, the spherical integration performed in Eq. 3 will not yield a correct coordination number. However, integrating the shell contributions in Fig. 3 results in the exact coordination number obtained from counting neighbors by shared faces of the solute and solvent polyhedra.

Employing Voronoi decomposition, Haberler and Steinhauser confirmed a surplus of C$_{2}\textit {mim}^{+}$ in the first solvation shell of various proteins despite the positive net charge of the protein (Haberler and Steinhauser 2011; Haberler et al. 2011). This is very likely due to expulsion of the cations from the strong anion-water network, as also observed in other studies (Schröder et al. 2007, 2009; Haberler and Steinhauser^2011^). Interestingly, the mean residence time of the anions at the protein surface is longer compared to the cations (Haberler and Steinhauser 2011) which can be explained by stronger interactions with the oppositely charged amino acid (Schröder 2017; Jaeger and Pfaendtner 2013; Klähn et al. 2011a; Micaêlo and Soares 2008).

#### Kirkwood-Buff theory

Another approach utilizing radial distribution functions is the so-called Kirkwood-Buff theory, which relates structural properties of the solutions

4 $$ G_{ij}(R) = {\int\limits_{0}^{R}} \left( g_{ij}(r)-1 \right) 4 \pi r^{2} dr, $$

to thermodynamic quantities (Kirkwood and Buff 1951). The Kirkwood-Buff integral is often interpreted as excess volume around a solute species when compared to an ideal solution. It is also possible to compute Kirkwood-Buff integrals of distinct solvation shells based on Voronoi tessellation (Zeindlhofer et al. 2018).

As a standard notation, $i,j = 1 {\ldots } 3$ denote water, the biomolecular solute, and the co-solvent (in our case the ionic liquid), respectively. Distance-dependent binding coefficients *ν*_23_(*R*) between the solute $i = 2$ and the ionic liquid $j = 3$ can be obtained as follows:

5 $$\begin{array}{@{}rcl@{}} \nu_{23}(R) &=& \frac{1}{n\pm}\left( \rho_{3}\left( G_{23}(R) - G_{21}(R) \right)-\frac{q^{\text{solute}}}{e}\right) \end{array} $$

6 $$\begin{array}{@{}rcl@{}} &=&\frac{1}{n\pm} \left( n_{23}(R)-\frac{\rho_{3}}{\rho_{1}}n_{21}(R)-\frac{q^{\text{solute}}}{e}\right) \end{array} $$

using coordination numbers defined in Eq.3 as well as the Donnan equilibrium condition (Pierce et al. 2008; Smith 2004; Smiatek 2017). Here ${q^{\text {solute}}}$ corresponds to the charge of the biomolecule in units of the elementary charge $e = 1.6022 \times 10^{-19}$ A s and $n{\pm }$ equals the number of indistinguishable ion species. The binding coefficient determines the transfer free energy (Smiatek 2017).

7 $$ F_{23}(R) = - k_{B} T \cdot \nu_{23}(R) $$

which estimates the excess energy at a given temperature *T* that is needed to transfer the ionic liquid from infinite distance to close proximity around the biomolecule.

In a MD study of a small $\beta $-hairpin peptide in aqueous [C_2_mim][OAc], where both native and denaturated conformations of the peptide were simulated, Kirkwood-Buff preferential binding coefficients revealed that the unfolded conformation attracts more anions while no difference in cation affinity is observed (Lesch et al. 2015). This agrees with the fact that the anionic influence on the denaturation process is stronger (Klähn et al. 2011a). The unfolding effect of [C_2_mim][OAc] on the peptide was also confirmed by experiment (Patel et al. 2014). In a follow-up publication by the same group, a similar approach was used to study the behavior of the same $\beta $-hairpin peptide in aqueous [C_2_mim][BF_4_] and [C_2_mim]Cl. The binding strength order of the anions was identified as OAc^−^≫ BF$_{4}^{-} >$ Cl^−^, and again it was found that OAc^−^ strongly binds to the denatured state, which is not observable for BF$_{4}^{-}$ and Cl^−^. The authors suggested that the larger acetate anion exhibits a conformation-dependent binding behavior due to specific interactions with the protein (Diddens et al. 2017). Furthermore, they found only negligible dependence of the binding behavior of C$_{2}\textit {mim}^{+}$ on the anion.

The interactions of caffeine, gallic acid, protocatechuic acid, and quercetin with aqueous mixtures of the ionic liquid [C_2_mim][OAc] were studied by a shell-resolved approach using Voronoi tessellation. As shown in the respective publications (Zeindlhofer *et al*. 2017, 2018), the investigated biomolecules are flat, aromatic systems of high anisotropy, which makes Voronoi tessellation necessary to distinguish between several solvation shells. Since Voronoi tessellation allows straightforward calculation of solvent shell volumes, a shell-wise calculation of Kirkwood-Buff interaction parameters analogous to Kirkwood-Buff integrals (Zeindlhofer et al. 2018) can be computed. However, the comparison of Kirkwood-Buff interaction parameters between solutes of different size is complicated, since they depend on the solute size. In contrast, the shell-wise potential of mean force (Zeindlhofer et al. 2017) measures solvent affinities of solutes differing in size via the ratio of shell to bulk concentration of a species.

### Solvation dynamics

**Fig. 4:**
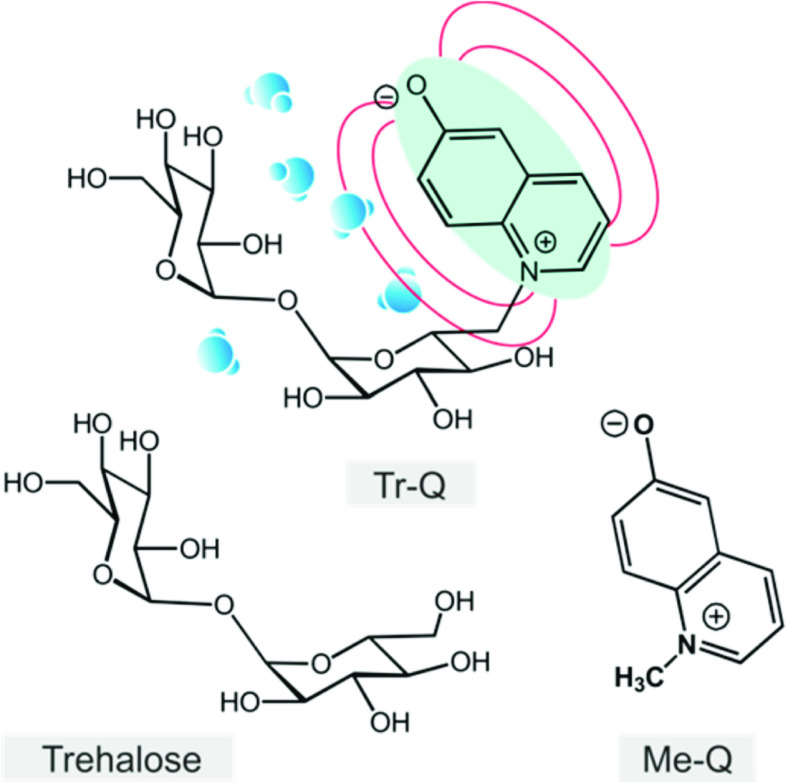
The chromophore oxyquinolinium betaine can be directly linked to the biomolecule of interest. Upon laser excitation, the local electric field (red lines) is changed and the response of the solvent molecules in the direct vicinity of the trehalose can be studied (Sajadi et al. 2014). Copyright 2014, American Chemical Society

The role of ionic liquids in solvation dynamics (Mandal and Samanta 2005; Samanta 2010; Roy and Maroncelli 2012; Terranova and Corcelli 2013; Liang et al. 2014; Heid and Schröder 2018) as well as the solvation dynamics of biomolecules (Sajadi et al. 2014; Heid and Schröder 2016; 2017) has attracted significant interest over the past decade. The time-dependent Stokes shift

8 $$ S(t) = \frac{\nu(t)-\nu(\infty)}{\nu(0)-\nu(\infty)} $$

monitors the transient behavior of the solvent relaxation after an electronic excitation of a dissolved chromophore. THz absorption bands of biomolecular hydration layers are generally swamped by absorption from bulk solvent. However, linking the chromophore to the biomolecule, the solvation properties of the immediate solvent molecules can be experimentally studied (Sajadi et al. 2014) as shown for the chromophore oxyquinolinium betaine attached to trehalose in Fig. 4.

In MD simulations, this experiment can be modeled by equilibrium (Roy and Maroncelli 2012; Schmollngruber et al. 2013; Terranova and Corcelli 2013) and non-equilibrium simulations (Heid and Schröder 2016, 2017, 2018). However, the latter approach seems to be more appropriate (Heid and Schröder 2018). The change of the Coulomb interaction energy ${\Delta } U^{elec}(t)$ of the chromophore atoms *j* with the solvent atoms *i*

9 $$ {\Delta} U^{elec}(t) = \frac{1}{4 \pi \epsilon_{0}} \sum\limits_{j} \sum\limits_{i} \frac{{\Delta} q_{j} \cdot q_{i}}{r_{ij}(t)} $$

relaxes during the non-equilibrium simulation after switching the partial charges $q_{j}$ of the solute from ground to excited state values:

10 $$ S(t)= \frac{{\Delta} U^{elec}(t)-{\Delta} U^{elec}(\infty)}{{\Delta} U^{elec}(0)-{\Delta} U^{elec}(\infty)} $$

In contrast to neutral liquids like water, ionic liquids can also respond to the change in the local electric field via translation (Terranova and Corcelli 2013; Schmollngruber et al. 2013), in particular the anions. The response of induced solvent dipoles is restricted to the first solvation shell (Schmollngruber et al. 2013). Using polarizable non-equilibrium molecular dynamics simulations, not only the normalized Stokes shift relaxation function but also the pure shift ${\Delta } U^{elec}(t)-{\Delta } U^{elec}(\infty )$ can be reproduced (Heid and Schröder 2018).

## Large flexible proteins

One of the “Technology Triggers” in Fig. 1 for protein stability was published in 2000 by Summers et al. who showed that ethylammonium nitrate can successfully refold denatured egg-white lysozyme (Summers and Flowers 2000). Several groups observed that ionic liquids can significantly stabilize certain proteins and enzymes (Lozano et al. 2001; Fujita et al. 2007; Weingärtner et al. 2012), especially lipases (Kaar et al. 2003; Ulbert et al. 2005; Lai et al. 2011), even at high temperatures (Ulbert et al. 2005). In some cases, enzyme activity, enantioselectivity, and regioselectivity in ionic liquids are increased compared to conventional organic solvents (Schöfer et al. 2001) as discussed in more detail in several reviews (Kragl et al. 2002; Park and Kazlauskas 2003; Yang and Pan 2005; van Rantwijk and Sheldon 2007; Moniruzzaman et al. 2010; Lai et al. 2011; Naushad et al. 2012; Gao et al. 2015; Chen et al. 2010; Zhao 2010; Weingärtner et al. 2012; Patel et al. 2014; Benedetto and Ballone 2016; Smiatek 2017; Schröder 2017; Zhao 2016).

**Fig. 5:**
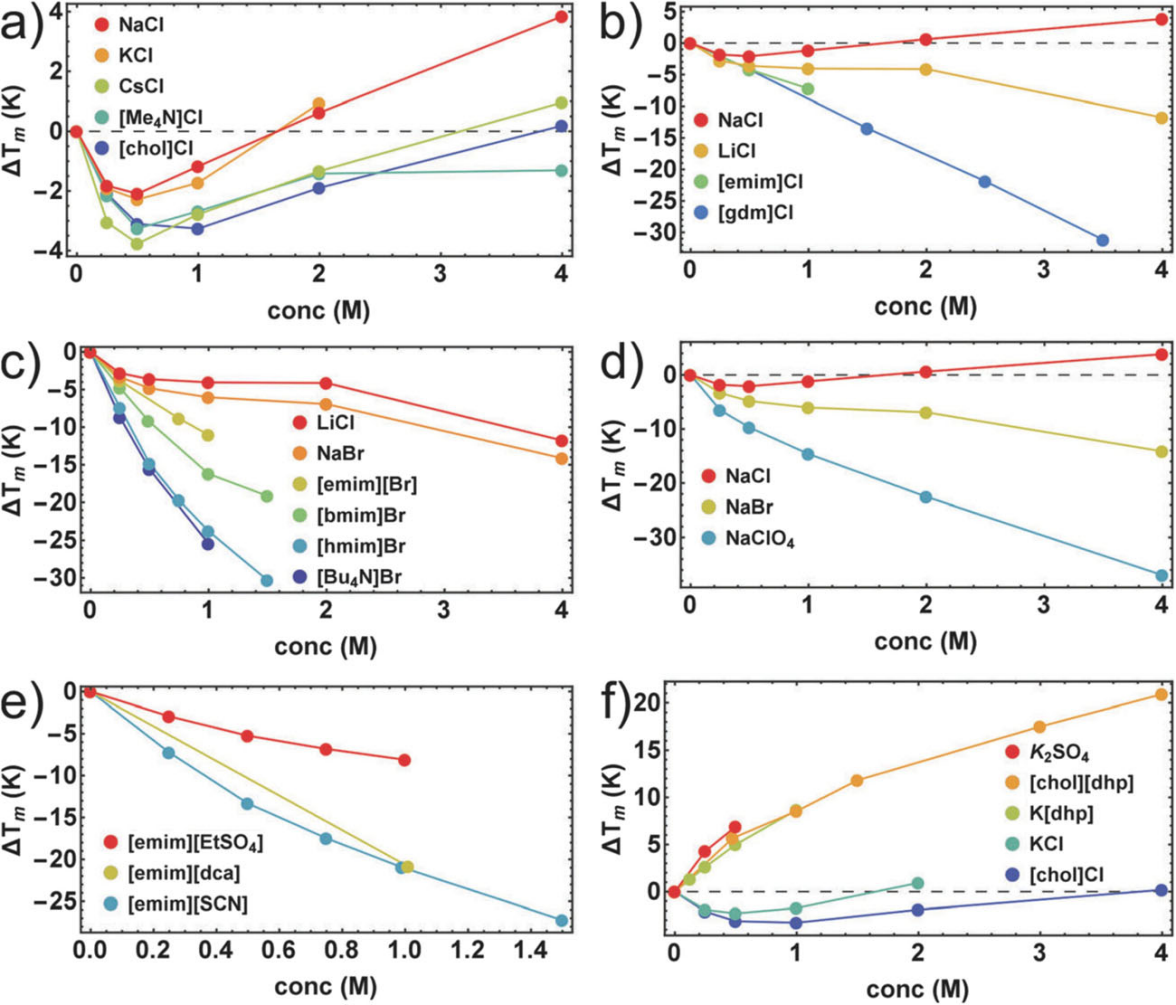
Salt-induced shifts of the melting temperature Δ*T*_*m*_ of RNase in aqueous ionic liquid mixtures with various ion concentrations (Senske et al. 2016)—Published by the PCCP Owner Societies. (**a**,**b**) chlorides (**c**) bromides (**d**) sodium salts (**e**) C_2_mim based salts (**f**) stabilizing oxo-anions

An observation that many groups agree with is that the presence of water in the ionic liquid is crucial for protein stability (Klähn et al. 2011a; Lozano et al. 2001). It was also observed that the effect of the anion on protein structure is more pronounced than the effect of the cation in most cases (Weingärtner et al. 2012; Constantinescu et al. 2007; Kaar et al. 2003). Furthermore, the destabilizing effect of ionic liquids in the aqueous mixture is a function of the ion concentration (Senske et al. 2016) as shown in Fig. 5. Stabilizing effects (Δ*T*_*m*_ > 0) were only observed for dihydrogenphosphate salts as well as sodium and potassium chloride above 2 M. Here, “enlightenment” from MD simulations on the mechanism is highly welcome.

In the quest of rationalizing ionic liquid protein interactions, MD simulations provide a useful tool for investigations at a molecular level and can complement experimental findings (Haberler and Steinhauser 2011). Although the first MD simulations of proteins were reported in the late 1970s (McCammon et al. 1977), the first MD simulation of a protein in an aqueous ionic liquid was published only in 2008 (Micaêlo and Soares 2008) revealing the time delay of computational analysis in Fig. 1 concerning interaction of proteins with ionic liquids. In Micaêlo and Soares (2008), investigated the behavior of the serine protease cutinase in [C_4_mim][PF_6_] and [C_4_mim][NO_3_] at various water contents. For [C_4_mim][PF_6_], 5–10% of water was necessary for optimal enzyme stabilization (Micaêlo and Soares 2008) indicating the importance of even small amounts of water.

A subsequent publication by the same group further investigated the influence of the protein surface characteristics on stability in ILs by conducting simulations of *Candida rugosa* lipase and *Bos taurus*$\alpha $-chrymotopsine with modified surface charges (Burney et al. 2015). By replacing surface lysines with glutamate, artificial mutations with positive-to-negative surface charge ratios were examined in simulation. On a 50-ns timescale, no stability difference for the different enzymes was found, but the authors explicitly state that such a timescale is likely too short (Burney et al. 2015). Due to the slow dynamics brought about by the high viscosity of ionic liquids, the need for large timescales when conducting simulations in ionic liquids is one of the great obstacles, and unfolding times are often too large to be accessible by simulation (Tung and Pfaendtner 2016). The problem of insufficient sampling in pure ionic liquids is also mentioned by Burney and Pfaendtner in their study on the behavior of *Candida rugosa* lipase in [C_4_mim][NO_3_] and [C_4_mim][PF_6_]. Despite sampling times of more than 100 ns, no visible effects of the ionic liquid on the protein could be observed, despite experimental evidence that the enzyme is heavily denatured in [C_4_mim][NO_3_] (Burney et al. 2015). Tung and Pfaendtner address this problem of large timescales by introducing an enhanced sampling method called “infrequent metadynamics,” an algorithm that allows estimation of unfolding times on a greatly accelerated timescale (Tung and Pfaendtner 2016). For assessing protein stability, several structural quantities are accessible via MD simulations:

### Radius of gyration

As unfolding increases protein volume, the loss of secondary structure leads to an increase of the radius of gyration $\mathrm {r_{gyr}}(t)$

11 $$ r_{gyr}(t) = \sqrt{\frac{\sum\limits_{i} m_{i} \left( \mathbf{r}_{i}(t) - \mathbf{r}_{\text{CM}}(t) \right)^{2}}{M}} $$

calculated with respect to the current center of mass $\mathbf {r}_{\text {CM}}(t)$ of the protein, where $m_{i}$ is the mass of the *i* th atom and *M* the total mass of the protein. Klähn et al. (2011a) reported that $\mathrm {r_{gyr}}(t)$ of *Candida antarctica* lipase B (CAL-B) in aqueous IL mixtures (hydration level 20% *w*/*w*) increased by 0.8, 1.1, and 2.8 Å for [C_4_mim][PF_6_], [C_4_mim][BF_4_], and [C_4_mim][NO_3_], respectively. They suggested that the strength of interactions between anions and enzyme is mainly governed by ion density and ion surface charge, favoring interactions with smaller ions which can form stronger hydrogen bonds with the protein. This finding goes along with the denaturation capabilities of classical ILs in Fig. 5e.

However, Chaban and co-workers (Chevrot et al. 2015) showed that the radius of gyration does not increase in ionic liquids with amino acid-based anions during the simulation period of 10 ns which may not be long enough to monitor the unfolding process due to the high viscosity of these ionic liquids. Interestingly, Shao (2013) reported that *r*_*gyr*_ of the B domain of protein A from *Staphylococcus aureus* actually decreased with increasing [C_4_mim]Cl content of the aqueous solution. Shao performed three independent simulations of 200 ns length to get meaningful values. A decrease of the protein radius was also observed by Bhattacharyya and co-workers (Ghosh et al. 2015) for lysozyme in [C_5_mim]Br. However, they computed the radius of hydration from the Stokes-Einstein relation:

12 $$ D = \frac{k_{B} T}{6 \pi \eta r_{H}} $$

using experimental diffusion coefficients from fluorescence correlation spectroscopy and the experimental viscosity $\eta $. The radius of gyration and the radius of hydration are linked by a factor of $\sqrt {5/3}$ (Schröder et al. 2015).

### Root-mean-squared displacements

The time-dependent root-mean-square deviation *RMSD* (Kufareva and Abagyan 2012)

13 $$ RMSD =\left \langle \sqrt{\frac{1}{N} {\sum\limits_{i}^{N}} \left( \mathbf{r}_{i}(t) - \mathbf{r}_{i}^{\text{ref}}\right)^{2}} \right\rangle_{t} $$

is defined as a function of the deviation of the current atomic coordinates $\mathbf {r}_{i}(t)$ from the coordinates $\mathbf {r}_{i}^{\text {ref}}$ of a reference structure with *N* atoms, usually at the beginning of the simulation or the native configuration.

In the first MD simulation of a protein in an ionic liquid by Micaêlo and Soares mentioned above, the authors analyzed the *RMSD* of cutinase in [C_4_mim][PF_6_] and [C_4_mim][NO_3_] at different hydration levels and temperatures (Micaêlo and Soares 2008). In accordance with experimental findings reporting that many enzymes retain activity in ILs with PF$_{6}^{-}$ anions and lose activity in ILs with NO$_{3}^{-}$ anions (Lozano et al. 2001; Kaar et al. 2003; Persson and Bornscheuer 2003; Lou et al. 2006), they observed generally larger *RMSD* values in [C_4_mim][NO_3_]. In contrast, Burney et al. did not find a significant change in the *RMSD* of *Candida rugosa* lipase 1 in [C_4_mim][PF_6_] and [C_4_mim][NO_3_] (Burney and Pfaendtner 2013). Pfaendtner and co-workers (Burney et al. 2015) demonstrated that the *RMSD* is not only a function of the ionic liquid but also depends on the nature of the protein. For the enzyme *Candida rugosa* lipase 1, the stability decreases in the following order of the solvent: water $>$ [C_4_mim]Cl $>$ [C_2_mim][EtSO_4_]. However, no *RMSD* trend is visible for *Bos taurus* $\alpha $*-chymotrypsin* in the very same solvents (Burney et al. 2015).

The stronger destabilizing effect of nitrate compared to hexafluorophosphate was confirmed by Klähn et al. (2011a) for CAL-B in various aqueous ionic liquid mixtures by means of *RMSD*-values and agreed well with the concurrent observation of an increased radius of gyration $r_{gyr}(t)$ mentioned in the last section. Although to less extent than the anions, longer alkyl chains lead to higher values of these quantities, indicating destabilization (Klähn et al. 2011b). Replacing butyl with a methoxyethyl group in C$_{4}\textit {mim}^{+}$ also resulted in substantial protein destabilization, possibly by the increased hydrogen-bonding ability of the methoxyethyl group.

**Fig. 6:**
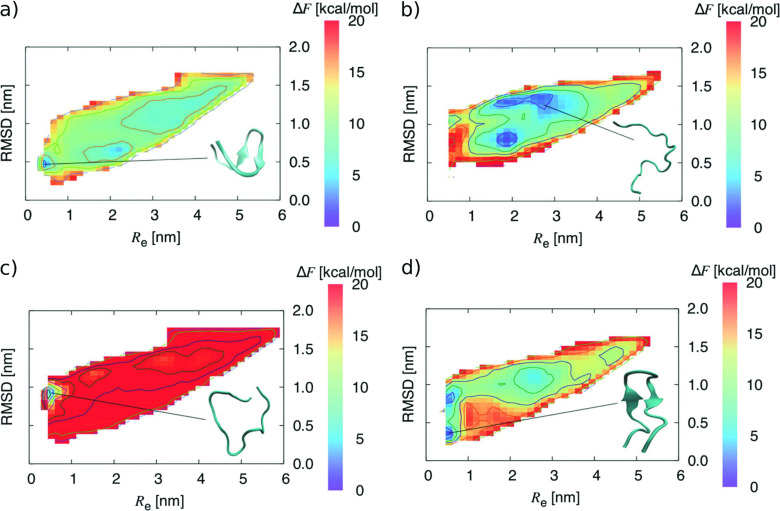
Free energy landscape with global energetic minimum conformation of a *β*-hairpin. **a** Native state of the peptide in water. **b** Denaturated state in aqueous [C_2_mim]. **c** Denaturated state in [C_2_mim]. **d** Denaturated state in [C_2_mim]Cl. *R*_*e*_ is the end-to-end distance of the peptide. (Diddens et al. 2017) - Published by the PCCP Owner Societies

Diddens et al. (2017) analyzed the *RMSD* of a $\beta $-hairpin peptide by metadynamics simulations in aqueous mixtures of [C_2_mim][OAc], [C_2_mim][BF_4_], and [C_2_mim]Cl. The $\beta $-hairpin peptide unfolded in aqueous mixture of [C_2_mim][OAc] and [C_2_mim][BF_4_] as visible in the insets of Fig. 6. However, the global energetic minima of the two unfolded configurations in Fig. 6b and c are completely different. The authors argued that the strong denaturating acetate attacks the intramolecular hydrogen bonds of the peptide. As a result, the peptide structure unfolds completely. In aqueous [C_2_mim][BF_4_], the global minimum is still located near that of the pure water solution; however, the *RMSD* is quite large. Since the anions are excluded from the surface of the peptide, this effect was attributed to the impact of the cations.

In contrast to the aqueous mixture of [C_2_mim][OAc] and [C_2_mim][BF_4_], the $\beta $-hairpin peptide did not unfold in aqueous [C_2_mim]Cl. The minimum structure of the peptide resembles the structure found in pure water (see Fig. 6a and d). This finding is in agreement with the experiment in Fig. 5b, where [C_2_mim]Cl was characterized to be only a weak denaturant.

In MD simulations of Shao (2013), the *RMSD* values of the B domain of protein A from *Staphylococcus aureus* decreased with increasing content of [C_4_mim]Cl which was interpreted as a stabilizing effect that may also be an effect of the increased viscosity.

### Root-mean-squared fluctuations

The root-mean-squared fluctuation

14 $$ RMSF(i) = \sqrt{ \frac{1}{t} {\sum\limits_{0}^{t}} \left( \mathbf{r}_{i}(t) - \langle \mathbf{r}_{i}\rangle_{t} \right)^{2}} $$

measures the fluctuation of atom *i* around its mean position $\langle \mathbf {r}_{i}\rangle _{t}$ (Pitera 2014). Experimentally, atomic flexibility is described with the crystallographic B-factor (Bornot et al. 2011). Amino acids with high *RMSF* values have an increased mobility which may also be an indicator for interactions with the ionic liquid (Attri et al. 2015; Burney and Pfaendtner 2013; Jaeger and Pfaendtner 2013) which breaks or at least disturbs intramolecular hydrogen bonding of the biomolecule.

For the analysis of simulations of three different cellulases in aqueous mixtures of the ionic liquid [C_2_mim][OAc], Jaeger et al. (2015) used the *RMSD* and an *RMSF* (averaged by residue) of the C_*α*_ atoms of the protein. They found that the three investigated cellulases (a cellulase from *Thrichderma viride*, a cellulase 5A from *Thermotoga maritima,* and an endoglucanase from *Pyrococcus horikoshii*) exhibited remarkably different stabilities in the aqueous ionic liquid [C_2_mim][OAc], highlighting that specific features of the protein, such as surface characteristics, and not only the ionic liquid itself determine stability.

### Electrostatic interactions

Non-bonded energies in MD simulation are most generally calculated by the electrostatic energy $U^{\text {elec}}(t)$

15 $$ U^{\text{elec}}(t) = \frac{1}{4 \pi \epsilon_{0}} \sum\limits_{i} \sum\limits_{j} \frac{q_{i} \cdot q_{j}}{r_{ij}(t)}  $$

between the partial charges $q_{i}$ and $q_{j}$ of the atoms *i* and *j* at a distance of $r_{ij}(t)$ as well as van-der-Waals energies $U^{\text {vdW}}(t)$

16 $$ U^{\text{vdW}}(t) = \sum\limits_{i} \sum\limits_{j} 4 \epsilon_{ij} \left( \left( \frac{\sigma_{ij}}{r_{ij}(t)} \right)^{12} - \left( \frac{\sigma_{ij}}{r_{ij}(t)}\right)^{6} \right) $$

based on the interatomic Lennard-Jones parameter $\sigma _{ij}$ and $\epsilon _{ij}$.

Usually, the first atomic index *i* concerns all atoms in the biomolecule *P* under investigation. The second atomic index *j* may also comprise all atoms (separated by at least three bonds) in the very same molecule to obtain intramolecular energies $U^{\text {elec}}_{\text {PP}}(t)$ and $U^{\text {vdW}}_{\text {PP}}(t)$. Steinhauser and co-workers (Haberler and Steinhauser 2011) computed $\langle U^{\text {elec}}_{\text {PP}} \rangle $ and $\langle U^{\text {vdW}}_{\text {PP}}\rangle $ for a zinc finger protein in aqueous solutions of [C_2_mim][CF_3_*SO*_3_], and found a rise in $\langle U^{\text {elec}}_{\text {PP}} \rangle (x_{\text {IL}})$ with increasing ionic mole fraction $x_{\text {IL}}$, whereas $\langle U^{\text {vdW}}_{\text {PP}} \rangle (x_{\text {IL}})$ decreased with increasing mole fraction. Moreover, the sum of electrostatic and van-der-Waals energies exhibited a maximum at an IL mole fraction of $x_{\text {IL}}= 0.073$. The authors termed this mole fraction a “magic point” since other secondary structure descriptors also showed extrema at this point. This behavior can be attributed to a change from dipolar screening by water to charge screening by the ionic liquid ions when the IL mole fraction increases, which is consistent with a rise of $\langle U^{\text {elec}}_{\text {PP}} \rangle (x_{\text {IL}})$.

Klähn et al. analyzed the electrostatic and van-der-Waals-interaction energy of hydrated *Candida antarctica* lipase B (CAL-B) with a set of ionic liquids composed of imidazolium- and guadinium-based cations and nitrate, tetrafluoroborate or hexfluorophosphate anions (Klähn et al. 2011a, 2011b). Here, the second atomic index *j* of Eq. 15 contains all solvent atoms of the particular solvent species. They found that interaction energies are dominated by Coulombic interactions $\langle U^{\text {elec}}_{\text {PA}} \rangle $ between the anions (A) and the protein, while smaller cation (C) contributions stem from van-der-Waals-interactions $\langle U^{\text {vdW}}_{\text {PC}} \rangle $. The charge on cations is more delocalized while the charge density on the smaller anions is higher, allowing them to form stronger hydrogen bonds with the protein. In their study on xylanase II in [C_2_mim][EtSO_4_] and [C_2_mim][OAc], Jaeger and Pfaendtner also attributed the low affinity of cations for negatively charged residues to the high charge delocalization (Jaeger and Pfaendtner 2013). Klähn et al. reported the highest values of 〈*U*^vdW^〉 + 〈*U*^elec^〉 (strongest hydrogen-bond interactions) for the NO$_{3}^{-}$ anion, which is consistent with experimental and simulation results that NO$_{3}^{-}$ destabilizes CAL-B (Klähn et al. 2011a; Micaêlo and Soares 2008). Weakest interactions were observed for the PF$_{6}^{-}$ anion, which stabilizes CAL-B. From experiment, it is also known that [C_4_mim][NO_3_] can dissolve solid CAL-B, while [C_4_mim][PF_6_] cannot (Klähn et al. 2011a; Lau et al. 2004).

### Solvation free energy

The general procedure of solvation free energy calculation is briefly described in the next section on small molecules. As already mentioned before, the simulations of proteins in ionic liquids are hampered by the increased viscosity in ionic liquids, requiring long simulation times for sufficient sampling (Tung and Pfaendtner 2016). Under these circumstances, it is impossible to acquire sufficient sampling for the calculation of solvation free energies (Klähn et al. 2011a). However, some other methods for obtaining thermodynamic information about proteins in ionic liquids have been employed. Klähn et al. (2011a) calculated the solvation free enthalpy $H_{\text {solv}}$ of CAL-B based on a linear response formalism (Klähn et al. 2011a),

17 $$ H_{\text{solv}} = \frac{1}{N^{\text{solute}}} \left( U_{\text{solv}} - \left( U^{\text{solute}}_{\text{gas}} + U^{\text{solvent}} \right) + P {\Delta} V \right),  $$

where $U_{\text {solv}}$ is the potential of the system containing $N^{\text {solute}}$ solvated solutes in $N^{\text {solvent}}$ solvent molecules, and $U^{\text {solute}}_{\text {gas}}$ and $U_{\text {solvent}}$ are the potential energies of the *N* solutes in gas phase and the pure solvent, respectively. The pressure-volume term is small and may be neglected, and when $N^{\text {solute}}= 1$ is assumed, Eq. 17 may be written as

$$\begin{array}{@{}rcl@{}} H_{\text{solv}} &=& U^{\text{solute}}_{\text{solv}} + U^{\text{solvent}}_{\text{solv}} + U^{\mathrm{solute-solvent}} \end{array} $$

18 $$\begin{array}{@{}rcl@{}} &&- \left( U^{\text{solute}}_{\text{gas}} + U^{\text{solvent}} \right) \end{array} $$

19 $$\begin{array}{@{}rcl@{}} &=& {\Delta} U_{\text{cage}} + {\Delta} U^{\text{solute}} + U^{\mathrm{solute-solvent}}. \end{array} $$

Here, $U^{\text {solute}}_{\text {solv}}$ is the potential energy of only the solute in solvent, $U^{\text {solvent}}_{\text {solv}}$ is the potential energy of only the solvent in the system, and $U^{\mathrm {solute-solvent}}$ is the solute-solvent interaction energy. ${\Delta } U_{\text {cage}} = U^{\text {solute}}_{\text {solv}} - U^{\text {solvent}}$ is termed the cage formation energy and measures the energy required to form a solute cavity in the solvent. Δ*U*^solute^ = *U* solvsolvent − *U* gassolute is the change in internal energy of the solute upon insertion into solvent (Klähn et al. 2011a). It was found that solvation enthalpies in ILs increased noticeably in ionic liquids compared to water, which is in accordance with experimental observations that CAL-B is less soluble in ionic liquids compared to water. It was also observed that these high solvation enthalpies were mainly due to large cage formation energies. These large cage formation energies are due to the strong interactions between ionic liquid ions compared to the weaker interactions in water. An exception was the ionic liquid [C_4_mim][PF_6_], in which the cage formation energy was even lower than in water. Furthermore, upon transfer from water to IL, the internal energy of the protein increased, except in [C_4_mim][PF_6_], corresponding to the experimentally observed stability increase in [C_4_mim][PF_6_]. Another approach to estimate the free energy landscape of a protein is the use of metadynamics (Barducci et al. 2008), which was already mentioned in the context of a small $\beta $-hairpin protein whose unfolding free energy landscape in aqueous [C_2_mim][OAc], [C_2_mim][BF_4_], and [C_2_mim][Cl] was calculated via metadynamics (Lesch et al. 2015; Diddens et al. 2017). As mentioned earlier, the strong denaturation of the peptide by [C_2_mim][OAc] apparent from preferential binding parameters was also visible in the free energy landscapes.

## Small rigid drugs

Ionic liquids are of special interest for the extraction of compounds from biomass. They can dissolve a wide range of biomatrices, even materials such as cellulose (Pinkert et al. 2009; Wang et al. 2012; Zhu et al. 2006) or chitin (Qin et al. 2010), and can enhance solubility of hydrophobic compounds in aqueous solution by acting as hydrotropes (Cláudio et al. 2015) or surfactants.

From the computational point of view, these molecules are much more rigid compared to a large flexible protein. Consequently, observables concerning size and shape, e.g., the radius of gyration as well as root-mean-squared displacements and fluctuations, are of less use for the small biomolecular drugs dissolved in the aqueous mixtures of ionic liquids. Hence, particular interactions like $\pi $-*π*-stacking or hydrogen bonding attract more attention. Also, free energy calculations, which are not feasible for complete proteins, may help to understand the biomolecular solvation of smaller solutes. However, the current knowledge on the interaction of small biomolecules with ILs is not as far progressed compared to protein solvation.

### Hydrotropic theory

The hydrotropic effect describes the increased water solubility of hydrophobic organic compounds by the addition of a co-solvent (Shimizu and Matubayasi^2014^; Eastoe *et al*. 2011; Cláudio et al. 2015; Zeindlhofer *et al*. 2017, 2018) which usually consists of small, amphiphilic molecules (Booth et al. 2012). In principle, the following mechanisms are discussed:

1. The solute forms a stable complex with few hydrotropic molecules via hydrogen bonding or $\pi $-*π*-stacking. The complex has an increased polarity, hence increased solubility in water.
2. The hydrotrope assembles in micelle-like structures in which the solute can be accommodated.
3. The water-immiscible solute induces a particular structure of the hydrotrope in the aqueous solution. This new structure promotes the solubility of the solute.

Imidazolium-based ILs seem to be promising candidates for hydrotropy. Their charged, hydrophilic ring is accompanied by more or less hydrophobic alkyl side chains (Tan et al. 2009; Cláudio et al. 2015). We recently reported (Zeindlhofer et al. 2018) on the solvation of coffee ingredients in [C_2_mim][OAc] mixtures. The cations seem to repel water in general and also anions from the surface of the solute at higher concentration. Even in the second solvation shell determined by Voronoi tessellation, the concentration of water is below bulk density which argues against mechanism 1. Nevertheless, water still has access to the solute surface, even at higher concentrations, and micellar cationic structures are not observed at the surface. Also, the last hydrotropic hypothesis 3 could not be proven. The inclusion of acetate in the water network is usually not a problem and does not change the water structure in the proximity of the solute very much. However, a Voronoi shell-wise computation of the dielectric permittivities reveals a subtle transition from the solute permittivity to the permittivity of the bulk solution (Zeindlhofer et al. 2018).

Furthermore, hydrogen bonding between the water and the solute is not changed by the cations (as suggested by hypothesis 3) but in competition with the anions. Interestingly, the solvation efficiency drops from tosylate to chloride for gallic acid (Cláudio et al. 2015): Cl^−^ <[MeSO$_{4}^{-}$] $<$ [CF$_{3}\textit {SO}_{3}^{-}$] $<$ [N(CN)$_{2}^{-}$] $<$ [Tos^−^]. A similar ranking was found in vanillin. Although the cations are the most prominent species at the solute surface, particular anion interactions are very important for the solvation behavior of the ionic liquid, i.e., hydrogen bonding. Cationic $\pi $-*π*-stacking is of minor importance.

### Solute-solvent interactions and solute aggregation

The extraction of shikimic acid from star anise pods using [C_2_mim]-based ionic liquids with a set of different anions (PF$_{6}^{-}$, NTf$_{2}^{-}$, BF$_{4}^{-}$, Cl^−^, OTf^−^, OAc^−^) was reported in an experimental study (Zirbs et al. 2013). Accompanying MD simulations were performed to identify the relevant molecular interactions in the extraction process: No correlation of the experimental extraction yield with the coordination number or the contact surface obtained from simulations could be established (Zirbs et al. 2013). Coordination numbers around shikimic acid are higher for cations, but anions seem to interact stronger since they are able to form hydrogen bonds, and their residence time in the first solvation shell around shikimic acid is longer than for cations. The number of anion hydrogen bonds correlates with the extraction yield, highlighting the importance of the anions in the extraction process.

In agreement with electrostatic analysis of the IL ion with proteins (Klähn et al. 2011a; Klähn et al. 2011b), $\langle U^{\text {elec}}_{\text {SA}} \rangle $ of the anions (A) with the solute (S) is much larger compared to $\langle U^{\text {elec}}_{\text {SC}} \rangle $ of the cations (C) (Zeindlhofer et al. 2017; Tomé et al. 2012). Furthermore, the van-der-Waals interaction $\langle U^{\text {vdW}}_{\text {SC}} \rangle $ between cations and the solute exceeds the corresponding Coulomb interaction $\langle U^{\text {elec}}_{\text {SC}} \rangle $ for C$_{2}\textit {mim}^{+}$ at the surface of various coffee ingredients (Zeindlhofer et al. 2017). Cations are mainly found parallel to the aromatic solute rings in a stacked orientation, while anions preferred positions around the ring that allowed hydrogen bonding to ring substituents as visible from $\langle U^{\text {elec}}_{\text {SA}} \rangle $. The same finding was observed for five different amino acids (glycine, alanine, valine, isoleucine, glutamic acid) in aqueous solutions of [C_4_mim][NTf_2_] (Tomé et al. 2012).

**Fig. 7:**
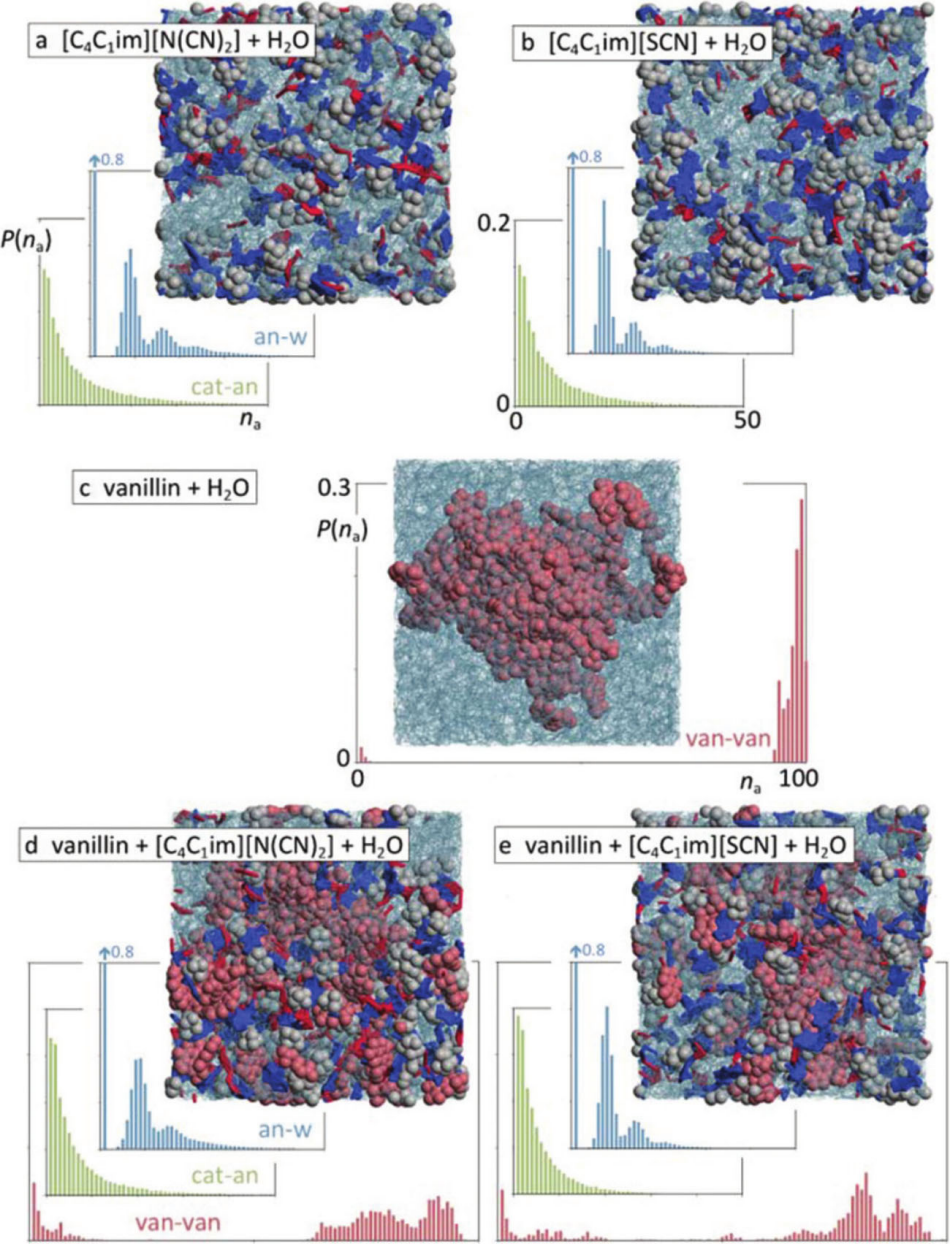
Distribution functions *P*(*n*_*a*_) of aggregate sizes in pure aqueous ionic liquid mixtures (**a**, **b**) and with vanillin (**d**, **e**). **c** depicts a vanillin-water mixture. Reproduced from Cláudio et al. (2015) with permission of The Royal Society of Chemistry

Another experimental study complemented by MD simulations concerned the extraction of vanillin in aqueous solutions of [C_4_mim][N(CN)_2_] and [C_4_mim][SCN] (Cláudio et al. 2015). In MD simulations, the ionic liquid ions form several filamentous ionic strands and interact with water in an anion-water hydrogen bond network. The hydrophobic vanillin molecules form a large droplet in pure water as visible in Fig. 7c, which is broken up into smaller aggregates in the aqueous IL solution by formation of larger vanillin-cation clusters and smaller vanillin-anion clusters in Fig. 7d and e.

### Solvation free energy

Enthalpic and entropic effects of the dissolution of a solute in a solvent can be best characterized by the computation of solvation free energies. In contrast to large proteins, the amount of sampling needed for these calculations is feasible for small compounds. A common approach is thermodynamic integration. During several simulations, a coupling parameter *λ* is stepwise increased from zero to one corresponding to a switching off of solute-solvent interactions $U^{\mathrm {solute-solvent}}$

20 $$ U(\lambda) = (1-\lambda) \; U^{\mathrm{solute-solvent}} + \lambda \cdot U^{\text{solvent}} $$

*U*^solvent^ corresponds to the total energy of the solvent only. The free energy difference between the initial and final $\lambda $-state is given by

21 $$ {\Delta} G_{\text{solv}} = \int \left\langle \frac{\partial U(\lambda)}{\partial \lambda} \right\rangle_{\lambda} d\lambda $$

To study molecular details of extraction of amino acids from aqueous solution using ILs (Absalan et al. 2010; Tomé et al. 2012), Seduraman et al. (2012) calculated the solvation free energy of L-tryptophan in pure and aqueous [C_4_mim][PF_6_], [C_8_mim][PF_6_], and [C_8_mim][BF_4_]. In contrast to experimental studies, they found only negligible differences in the free energies in the pure ILs. However, including residual water in the IL simulations, which is higher in [C_4_mim][PF_6_], the experimental trend is reproduced illustrating the importance of water for the extraction process. The solvation in BF$_{4}^{-}$-based ILs is more favorable, probably due to the fact that the water density is 7.5 times higher compared to the hexafluorophosphate systems. Furthermore, electrostatic interactions of tryptophan with BF$_{4}^{-}$ are stronger than with PF$_{6}^{-}$. This anion dependency was observed for other amino acids as well (Absalan et al. 2010).

## Conclusion

After the initial hype in the beginning of the twenty-first century, expectations on ionic liquids decreased because of issues with the price, viscosity, the stability in water, the toxicity, and biodegradability. Often, the unique selling point of ionic liquids was missing in applications to justify the higher cost compared to conventional solvents. Moreover, exorbitant promises during the hype prompted several authors to question the current status of knowledge and the usefulness of IL in general (Kunz and Häckl 2016). Consequently, to prevent making mistakes again, which were made during the hype, good practice routines should go without saying. In the current review, we briefly summarized meaningful computational methods to investigate the solvation of large and small biomolecules in aqueous ionic liquid mixtures.

The increasing number of publications concerning ionic liquids demonstrates their unbowed interest. However, in order to make real progress during the implementation of ionic liquids and their computational analysis, several ideas should be taken into account:

1. The vast majority of computational works deal with aprotic imidazolium-based ionic liquids. Maybe, protic imidazoliums as well as other cations like choline may be more biodegradable and cheaper. Therefore, juxtaposition of computational results of these “new” ionic liquids to the aprotic imidazolium-based ionic liquid are interesting.
2. The same is true for the anions. Tetrafluoroborate and hexafluorophosphate can easily be coarse-grained and much experimental data exists, but these anions are of less interest for the experimenter. Therefore, other anions such as dihydrogenphosphate (dhp), methyl- and ethylsulfate, hydrophobic bis-(trifluoromethylsulfonyl)-imide NTf$_{2}^{-}$, or hydrophilic dicyanamide N(CN)$_{2}^{-}$ should also be included in the portfolio of the theoreticians. Even deep eutectic solvents are interesting but currently still mostly neglected in MD simulations on solvation of biomolecules.
3. Many solvation effects depend on the concentration of the co-solvent and should be given much more room in current theoretical investigations as these simulations can be simply parallelized.
4. Particular effects of ionic liquids should be tested on more than one protein as the observation made may be a peculiarity of the investigated protein.
5. Polarizable force fields to include electronic effects may also be an option.
6. The computational setup is of utmost importance for the reliability of the MD results. Hence, simulation system sizes should be large enough to prevent artifacts from self-interactions. For example, at least five complete solvation shells around the biomolecule should be considered. The simulation period should be at least 50 ns in low viscous systems, i.e., mixtures with high water content, and even much longer with increasing ionic liquid concentration. Of course, the simulation period has also to be prolonged when analyzing folding/unfolding processes. Here, smaller proteins may be more useful than larger proteins to cover the complete process or at least major parts of it.

Applying these suggestions to future simulations on biomolecular solvation in ionic liquids should help to reach the “Plateau of Productivity” within reasonable time.

## References

[CR1] Absalan G, Akhond M, Sheikhian L (2010). Partitioning of acidic, basic and neutral amino acids into imidazolium-based ionic liquids. Amino Acids.

[CR2] Anderson JL, Ding J, Welton T, Armstrong DW (2002). Characterizing ionic liquids on the basis of multiple solvation interactions. J Am Chem Soc.

[CR3] Anugwom I, Mäki-Arvela P, Virtanen P, Willför S, Sjöholm R, Mikkola JP (2012). Selective extraction of hemicelluloses from spruce using switchable ionic liquids. Carbohydr Polym.

[CR4] Armand M, Endres F, MacFarlane DR, Ohno H, Scrosati B (2009). Ionic-liquid materials for the electrochemical challenges of the future. Nat Mater.

[CR5] Attri P, Sarinont T, Kim M, Amano T, Koga K, Cho AE, Ha Choi E, Shiratani M (2015). Influence of ionic liquid and ionic salt on protein against the reactive species generated using dielectric barrier discharge plasma. Sci Rep.

[CR6] Barducci A, Bussi G, Parrinello M (2008). Well-tempered metadynamics: a smoothly converging and tunable free-energy method. Phys Rev Lett.

[CR7] Benedetto A, Ballone P (2016). Room temperature ionic liquids meet biomolecules: a microscopic view of structure and dynamics. ACS Sustain Chem Eng.

[CR8] Bhargava BL, Klein ML (2009). Aqueous solutions of imidazolium ionic liquids: molecular dynamics studies. Soft Matter.

[CR9] Booth JJ, Abbott S, Shimizu S (2012). Mechanism of hydrophobic drug solubilization by small molecule hydrotropes. J Phys Chem B.

[CR10] Bornot A, Etchebest C, De Brevern AG (2011). Predicting protein flexibility through the prediction of local structures. Proteins.

[CR11] Brandt A, Gräsvik J, Hallett JP, Welton T (2013). Deconstruction of lignocellulosic biomass with ionic liquids. Green Chem.

[CR12] Burney P, Nordwald EM, Hickman K, Kaar JL, Pfaendtner J (2015). Molecular dynamics investigation of the ionic liquid/enzyme interface: application to engineering enzyme surface charge. Proteins: Struct Funct Bioinf.

[CR13] Burney P, Pfaendtner J (2013). Structural and dynamic features of Candida rugosa lipase 1 in water, octane, toluene, and ionic liquids BMIM-PF6 and BMIM-NO3. J Phys Chem B.

[CR14] Chang TM, Dang LX, Devanathan R, Dupuis M (2010). Structure and dynamics of N, N-Diethyl-N-methylammonium triflate ionic liquid, neat and with water, from molecular dynamics simulations. J Phys Chem A.

[CR15] Chen X, Liu J, Wang J (2010). Ionic liquids in the assay of proteins. Anal Methods.

[CR16] Chevrot G, Fileti EE, Chaban VV (2015). Enhanced stability of the model mini-protein in amino acid ionic liquids and their aqueous solutions. J Comput Chem.

[CR17] Cláudio AFM, Freire MG, Freire CS, Silvestre AJ, Coutinho JA (2010). Extraction of vanillin using ionic-liquid-based aqueous two-phase systems. Sep Purif Technol.

[CR18] Cláudio AFM, Neves MC, Shimizu K, Lopes JNC, Freire MG, Coutinho JAP (2015). The magic of aqueous solutions of ionic liquids: ionic liquids as a powerful class of catanionic hydrotropes. Green Chem.

[CR19] Constantinescu D, Weingärtner H, Herrmann C (2007). Protein denaturation by ionic liquids and the hofmeister series: a case study of aqueous solutions of ribonuclease–A. Angew Chem Int Ed.

[CR20] Cull S, Holbrey J, Vargas-Mora V, Seddon K, Lye G (2000). Room-temperature ionic liquids as replacements for organic solvents in multiphase bioprocess operations. Biotechnol Bioeng.

[CR21] Diddens D, Lesch V, Heuer A, Smiatek J (2017). Aqueous ionic liquids and their influence on peptide conformations: denaturation and dehydration mechanisms. Phys Chem Chem Phys.

[CR22] Dreyer S, Kragl U (2008). Ionic liquids for aqueous two-phase extraction and stabilization of enzymes. Biotechnol Bioeng.

[CR23] Earle MJ, Seddon KR (2009). Ionic liquids. Green solvents for the future. Pure Appl Chem.

[CR24] Eastoe J, Hatzopoulos MH, Dowding PJ (2011). Action of hydrotropes and alkyl-hydrotropes. Soft Matter.

[CR25] Fort DA, Remsing RC, Swatloski RP, Moyna P, Moyna G, Rogers RD (2007). Can ionic liquids dissolve wood? Processing and analysis of lignocellulosic materials with 1-n-butyl-3-methylimidazolium chloride. Green Chem.

[CR26] Freemantle M (1998). Designer solvents. Chem Eng News.

[CR27] Freire MG, Cláudio AFM, Araújo JMM, Coutinho JAP, Marrucho IM, Lopes JNC, Rebelo LPN (2012). Aqueous biphasic systems: a boost brought about by using ionic liquids. Chem Soc Rev.

[CR28] Fujita K, MacFarlane DR, Forsyth M, Yoshizawa-Fujita M, Murata K, Nakamura N, Ohno H (2007). Solubility and stability of cytochrome c in hydrated ionic liquids: effect of oxo acid residues and kosmotropicity. Biomacromolecules.

[CR29] Gao WW, Zhang FX, Zhang GX, Zhou CH (2015). Key factors affecting the activity and stability of enzymes in ionic liquids and novel applications in biocatalysis. Biochem Eng J.

[CR30] Ghosh S, Parui S, Jana B, Bhattacharyya K (2015). Ionic liquid induced dehydration and domain closure in lysozyme: FCS and MD simulation. J Chem Phys.

[CR31] Gupta KM, Hu Z, Jiang J (2013). Molecular insight into cellulose regeneration from a cellulose/ionic liquid mixture: effects of water concentration and temperature. RSC Adv.

[CR32] Gutowski KE, Broker GA, Willauer HD, Huddleston JG, Swatloski RP, Holbrey JD, Rogers RD (2003). Controlling the aqueous miscibility of ionic liquids: aqueous biphasic systems of water-miscible ionic liquids and water-structuring salts for recycle, metathesis, and separations. J Am Chem Soc.

[CR33] Haberler M, Steinhauser O (2011). On the influence of hydrated ionic liquids on the dynamical structure of model proteins: a computational study. Phys Chem Chem Phys.

[CR34] Haberler M, Schröder C, Steinhauser O (2011). Solvation studies of a zinc finger protein in hydrated ionic liquids. Phys Chem Chem Phys.

[CR35] Hallett JP, Welton T (2011). Room-temperature ionic liquids: solvents for synthesis and catalysis. 2. Chem Rev.

[CR36] Heid E, Schröder C (2016). Computational solvation dynamics of oxyquinolinium betaine linked to trehalose. J Chem Phys.

[CR37] Heid E, Schröder C (2017). Effect of a tertiary butyl group on polar solvation dynamics in aqueous solution: a computational approach. J Phys Chem B.

[CR38] Heid E, Schröder C (2018). Solvation dynamics in polar solvents and imidazolium ionic liquids: failure of linear response approximations. Phys Chem Chem Phys.

[CR39] Holbrey JD, Seddon KR (1999). Ionic liquids. Clean Technol Environ Policy.

[CR40] Itoh T (2017). Ionic liquids as tool to improve enzymatic organic synthesis. Chem Rev.

[CR41] Jaeger VW, Pfaendtner J (2013). Structure, dynamics, and activity of xylanase solvated in binary mixtures of ionic liquid and water. ACS Chem Biol.

[CR42] Jaeger V, Burney P, Pfaendtner J (2015). Comparison of three ionic liquid-tolerant cellulases by molecular dynamics. Biophys J.

[CR43] Kaar JL, Jesionowski AM, Berberich JA, Moulton R, Russell AJ (2003). Impact of ionic liquid physical properties on lipase activity and stability. J Am Chem Soc.

[CR44] Kelkar MS, Maginn EJ (2007). Effect of temperature and water content on the shear viscosity of the ionic liquid 1-ethyl-3-methylimidazolium bis(trifluoromethanesulfonyl)imide as studied by atomistic simulations. J Phys Chem B.

[CR45] Kilpeläinen I, Xie H, King A, Granstrom M, Heikkinen S, Argyropoulos DS (2007). Dissolution of wood in ionic liquids. J Agric Food Chem.

[CR46] King AWT, Asikkala J, Mutikainen I, Järvi P, Kilpeläinen I (2011). Distillable acid–base conjugate ionic liquids for cellulose dissolution and processing. Angew Chem Int Ed.

[CR47] Kirkwood JG, Buff FP (1951). The statistical mechanical theory of solutions. I. J Chem Phys.

[CR48] Klähn M, Lim GS, Wu P (2011a) How ion properties determine the stability of a lipase enzyme in ionic liquids: a molecular dynamics study. Phys Chem Chem Phys 13(41):18647–18660 10.1039/c1cp22056j 21947063

[CR49] Klähn M, Lim GS, Seduraman A, Wu P (2011b) On the different roles of anions and cations in the solvation of enzymes in ionic liquids. Phys Chem Chem Phys 13(4):1649–1662 10.1039/c0cp01509a 21132189

[CR50] Kragl U, Eckstein M, Kaftzik N (2002). Enzyme catalysis in ionic liquids. Curr Opin Biotechnol.

[CR51] Kufareva I, Abagyan R (2012). Methods of protein structure comparison. Methods Mol Biol.

[CR52] Kunz W, Häckl K (2016). The hype with ionic liquids as solvents. Chem Phys Lett.

[CR53] Lai JQ, Li Z, Lü YH, Yang Z (2011). Specific ion effects of ionic liquids on enzyme activity and stability. Green Chem.

[CR54] Lau RM, Sorgedrager MJ, Carrea G, Rantwijk FV, Secundo F, Sheldon RA (2004). Dissolution of Candida antarctica lipase B in ionic liquids: effects on structure and activity. Green Chem.

[CR55] Lee SY, Khoiroh I, Ooi CW, Ling TC, Show PL (2017). Recent advances in protein extraction using ionic liquid-based aqueous two-phase systems. Sep Purif Rev.

[CR56] Lesch V, Heuer A, Tatsis VA, Holm C, Smiatek J (2015). Peptides in the presence of aqueous ionic liquids: tunable co-solutes as denaturants or protectants?. Phys Chem Chem Phys.

[CR57] Liang M, Zhang X, Kaintz A, Ernsting NP, Maroncelli M (2014). Solvation dynamics in a prototypical ionic liquid + dipolar aprotic liquid mixture: 1-butyl-3-methylimidazolium tetrafluoroborate + acetonitrile. J Phys Chem B.

[CR58] Lou WY, Zong MH, Smith TJ, Wu H, Wang JF (2006). Impact of ionic liquids on papain: an investigation of structure–function relationships. Green Chem.

[CR59] Lozano P, de Diego T, Guegan JP, Vaultier M, Iborra JL (2001). Stabilization of $\alpha $α-chymotrypsin by ionic liquids in transesterification reactions. Biotechnol Bioeng.

[CR60] Mäki-Arvela P, Anugwom I, Virtanen P, Sjöholm R, Mikkola JP (2010). Dissolution of lignocellulosic materials and its constituents using ionic liquids—a review. Ind Crops Prod.

[CR61] Mandal PK, Samanta A (2005). Fluorescence studies in a pyrrolidinium ionic liquid: polarity of the medium and solvation dynamics. J Phys Chem B.

[CR62] McCammon JA, Gelin BR, Karplus M (1977). Dynamics of folded proteins. Nature.

[CR63] Micaêlo NM, Soares CM (2008). Protein structure and dynamics in ionic liquids. Insights from molecular dynamics simulation studies. J Phys Chem B.

[CR64] Mikkola JP, Kirilin A, Tuuf JC, Pranovich A, Holmbom B, Kustov LM, Murzin DY, Salmi T (2007). Ultrasound enhancement of cellulose processing in ionic liquids: from dissolution towards functionalization. Green Chem.

[CR65] Moniruzzaman M, Nakashima K, Kamiya N, Goto M (2010). Recent advances of enzymatic reactions in ionic liquids. Biochem Eng J.

[CR66] Naushad M, ALOthman ZA, Khan AB, Ali M (2012). Effect of ionic liquid on activity, stability, and structure of enzymes: a review. Int J Biol Macromol.

[CR67] Neumayr G, Rudas T, Steinhauser O (2010). Global and local Voronoi analysis of solvation shells of proteins. J Chem Phys.

[CR68] Omta AW, Kropman MF, Woutersen S, Bakker HJ (2003). Negligible effect of ions on the hydrogen-bond structure in liquid water. Science.

[CR69] Park S, Kazlauskas RJ (2003). Biocatalysis in ionic liquids—advantages beyond green technology. Curr Opin Biotechnol.

[CR70] Parviainen A, King AWT, Mutikainen I, Hummel M, Selg C, Hauru LKJ, Sixta H, Kilpeläinen I (2013). Predicting cellulose solvating capabilities of acid—base conjugate ionic liquids. ChemSusChem.

[CR71] Pârvulescu VI, Hardacre C (2007). Catalysis in ionic liquids. Chem Rev.

[CR72] Patel R, Kumari M, Khan AB (2014). Recent advances in the applications of ionic liquids in protein stability and activity: a review. Appl Biochem Biotechnol.

[CR73] Pei Y, Wang J, Wu K, Xuan X, Lu X (2009). Ionic liquid-based aqueous two-phase extraction of selected proteins. Sep Purif Technol.

[CR74] Pereira JFB, Lima ÁS, Freire MG, Coutinho JAP (2010). Ionic liquids as adjuvants for the tailored extraction of biomolecules in aqueous biphasic systems. Green Chem.

[CR75] Pereira JFB, Ventura SPM, e Silva FA, Shahriari S, Freire MG, Coutinho JAP (2013). Aqueous biphasic systems composed of ionic liquids and polymers: a platform for the purification of biomolecules. Sep Purif Technol.

[CR76] Persson M, Bornscheuer UT (2003). Increased stability of an esterase from Bacillus stearothermophilus in ionic liquids as compared to organic solvents. J Mol Catal B: Enzym.

[CR77] Pierce V, Kang M, Weerasinghe S, Smith PE, Aburi M (2008). Recent applications of Kirkwood-Buff theory to biological systems. Cell Biochem Biophys.

[CR78] Pinkert A, Marsh KN, Pang S, Staiger MP (2009). Ionic liquids and their interaction with cellulose. Chem Rev.

[CR79] Pitera JW (2014). Expected distributions of root-mean-square positional deviations in proteins. J Phys Chem B.

[CR80] Plechkova NV, Seddon KR (2008). Applications of ionic liquids in the chemical industry. Chem Soc Rev.

[CR81] Qin Y, Lu X, Sun N, Rogers RD (2010). Dissolution or extraction of crustacean shells using ionic liquids to obtain high molecular weight purified chitin and direct production of chitin films and fibers. Green Chem.

[CR82] Raut DG, Sundman O, Su W, Virtanen P, Sugano Y, Kordas K, Mikkola JP (2015). A morpholinium ionic liquid for cellulose dissolution. Carbohydr Polym.

[CR83] Roy D, Maroncelli M (2012). Simulations of solvation and solvation dynamics in an idealized ionic liquid model. J Phys Chem B.

[CR84] Sajadi M, Berndt F, Richter C, Gerecke M, Mahrwald R, Ernsting NP (2014). Observing the hydration layer of trehalose with a linked molecular terahertz probe. J Phys Chem Lett.

[CR85] Samanta A (2010). Solvation dynamics in ionic liquids: what we have learned from the dynamic fluorescence stokes shift studies. J Phys Chem Lett.

[CR86] Sasaki H (2015). Simulating hype cycle curves with mathematical functions : some examples of high-tech trends in Japan. Int J Inf Technol.

[CR87] Schmollngruber M, Schröder C, Steinhauser O (2013). Polarization effects on the solvation dynamics of coumarin C153 in ionic liquids: components and their cross-correlations. J Chem Phys.

[CR88] Schöfer SH, Kaftzik N, Wasserscheid P, Kragl U (2001). Enzyme catalysis in ionic liquids: lipase catalysed kinetic resolution of 1-phenylethanol with improved enantioselectivity. Chem Commun.

[CR89] Schröder C (2017) Proteins in ionic liquids: current status of experiments and simulations. Top Curr Chem 375(2) 10.1007/s41061-017-0110-2 PMC5480425 28176271

[CR90] Schröder C, Rudas T, Neumayr G, Benkner S, Steinhauser O (2007). On the collective network of ionic liquid/water mixtures. I. Orientational structure. J Chem Phys.

[CR91] Schröder C, Hunger J, Stoppa A, Buchner R, Steinhauser O (2008). On the collective network of ionic liquid/water mixtures. II. Decomposition and interpretation of dielectric spectra. J Chem Phys.

[CR92] Schröder C, Neumayr G, Steinhauser O (2009). On the collective network of ionic liquid/water mixtures. III. Structural analysis of ionic liquids on the basis of Voronoi decomposition. J. Chem. Phys.

[CR93] Schröder C, Sega M, Schmollngruber M, Gailberger E, Braun D, Steinhauser O (2014). On the collective network of ionic liquid/water mixtures. IV. Kinetic and rotational depolarization. J. Chem. Phys.

[CR94] Schröder C, Steinhauser O, Sasisanker P, Weingärtner H (2015) Orientational alignment of amyloidogenic proteins in pre-aggregated solutions. Phys Rev Lett 114(12). 10.1007/s41061-017-0110-210.1007/s41061-017-0110-2 10.1103/PhysRevLett.114.128101 25860772

[CR95] Seduraman A, Wu P, Klähn M (2012). Extraction of tryptophan with ionic liquids studied with molecular dynamics simulations. J Phys Chem B.

[CR96] Senske M, Constantinescu-Aruxandei D, Havenith M, Herrmann C, Weingärtner H, Ebbinghaus S (2016). The temperature dependence of the Hofmeister series: thermodynamic fingerprints of cosolute—protein interactions. Phys Chem Chem Phys.

[CR97] Shao Q (2013). On the influence of hydrated imidazolium-based ionic liquid on protein structure stability: a molecular dynamics simulation study. J Chem Phys.

[CR98] Shimizu S, Matubayasi N (2014). Hydrotropy: Monomer–Micelle equilibrium and minimum hydrotrope concentration. J Phys Chem B.

[CR99] Smiatek J (2017). Aqueous ionic liquids and their effects on protein structures: an overview on recent theoretical and experimental results. J Phys: Condens Matter.

[CR100] Smith PE (2004). Cosolvent interactions with biomolecules: relating computer simulation data to experimental thermodynamic data. J Phys Chem B.

[CR101] Steudte S, Neumann J, Bottin-Weber U, Diedenhofen M, Arning J, Stepnowski P, Stolte S (2012). Hydrolysis study of fluoroorganic and cyano-based ionic liquid anions—consequences for operational safety and environmental stability. Green Chem.

[CR102] Summers CA, Flowers RA (2000). Protein renaturation by the liquid organic salt ethylammonium nitrate. Protein Sci.

[CR103] Swatloski RP, Spear SK, Holbrey JD, Rogers RD (2002). Dissolution of cellose with ionic liquids. J Am Chem Soc.

[CR104] Tan SSY, MacFarlane DR, Upfal J, Edye LA, Doherty WOS, Patti AF, Pringle JM, Scott JL (2009). Extraction of lignin from lignocellulose at atmospheric pressure using alkylbenzenesulfonate ionic liquid. Green Chem.

[CR105] Terranova ZL, Corcelli SA (2013). On the mechanism of solvation dynamics in imidazolium-based ionic liquids. J Phys Chem B.

[CR106] Thuy Pham TP, Cho CW, Yun YS (2010). Environmental fate and toxicity of ionic liquids: a review. Water Res.

[CR107] Tomé LIN, Jorge M, Gomes JRB, Coutinho JAP (2012). Molecular dynamics simulation studies of the interactions between ionic liquids and amino acids in aqueous solution. J Phys Chem B.

[CR108] Tung HJ, Pfaendtner J (2016). Kinetics and mechanism of ionic-liquid induced protein unfolding: application to the model protein HP35. Mol Syst Des Eng.

[CR109] Ulbert O, Bélafi-Bakó K, Tonova K, Gubicza L (2005). Thermal stability enhancement of Candida rugosa lipase using ionic liquids. Biocatal Biotransform.

[CR110] van Rantwijk F, Sheldon RA (2007). Biocatalysis in ionic liquids. Chem Rev.

[CR111] Varela LM, Méndez-Morales T, Carrete J, Gómez-González V, Docampo-Álvarez B, Gallego LJ, Cabeza O, Russina O (2015). Solvation of molecular cosolvents and inorganic salts in ionic liquids: a review of molecular dynamics simulations. J Mol Liq.

[CR112] Ventura SPM, e Silva FA, Quental MV, Mondal D, Freire MG, Coutinho JAP (2017). Ionic-liquid-mediated extraction and separation processes for bioactive compounds: past, present, and future trends. Chem Rev.

[CR113] Ventura SPM, Neves CMSS, Freire MG, Marrucho IM, Oliveira J, Coutinho JAP (2009). Evaluation of anion influence on the formation and extraction capacity of ionic-liquid-based aqueous biphasic systems. J Phys Chem B.

[CR114] Wang H, Gurau G, Rogers RD (2012). Ionic liquid processing of cellulose. Chem Soc Rev.

[CR115] Wasserscheid P (2017) Talk at the 655th Heraeus symposium: Surfaces and Interfaces of Ionic Liquids

[CR116] Weingärtner H, Cabrele C, Herrmann C (2012). How ionic liquids can help to stabilize native proteins. Phys Chem Chem Phys.

[CR117] Welton T (1999). Room-temperature ionic liquids. Solvents for synthesis and catalysis. Chem Rev.

[CR118] Yang Z, Pan W (2005). Ionic liquids: green solvents for nonaqueous biocatalysis. Enzyme Microb Technol.

[CR119] Zeindlhofer V, Khlan D, Bica K, Schröder C (2017). Computational analysis of the solvation of coffee ingredients in aqueous ionic liquid mixtures. RSC Adv.

[CR120] Zeindlhofer V, Berger M, Steinhauser O, Schröder C (2018). A shell-resolved analysis of preferential solvation of coffee ingredients in aqueous mixtures of the ionic liquid 1-ethyl-3-methylimidazolium acetate. J Chem Phys.

[CR121] Zhang Y, Cremer P (2006). Interactions between macromolecules and ions: the Hofmeister series. Curr Opin Chem Biol.

[CR122] Zhang Y, Cremer PS (2009). The inverse and direct Hofmeister series for lysozyme. Proc Natl Acad Sci USA.

[CR123] Zhang J, Wu J, Yu J, Zhang X, He J, Zhang J (2017). Application of ionic liquids for dissolving cellulose and fabricating cellulose-based materials: state of the art and future trends. Mater Chem Front.

[CR124] Zhao H (2010). Methods for stabilizing and activating enzymes in ionic liquids—a review. J Chem Technol Biotechnol.

[CR125] Zhao H (2016). Protein stabilization and enzyme activation in ionic liquids: specific ion effects: protein stabilization and enzyme activation in ionic liquids. J Chem Technol Biotechnol.

[CR126] Zhu S, Wu Y, Chen Q, Yu Z, Wang C, Jin S, Ding Y, Wu G (2006). Dissolution of cellulose with ionic liquids and its application: a mini-review. Green Chem.

[CR127] Zirbs R, Strassl K, Gaertner P, Schröder C, Bica K (2013). Exploring ionic liquid-biomass interactions: towards the improved isolation of shikimic acid from star anise pods. RSC Adv.

